# Dissecting the Structural and Conductive Functions of Nanowires in *Geobacter sulfurreducens* Electroactive Biofilms

**DOI:** 10.1128/mbio.03822-21

**Published:** 2022-02-15

**Authors:** Yin Ye, Xing Liu, Kenneth H. Nealson, Christopher Rensing, Shuping Qin, Shungui Zhou

**Affiliations:** a Fujian Provincial Key Laboratory of Soil Environmental Health and Regulation, College of Resources and Environment, Fujian Agriculture and Forestry University, Fuzhou, China; b Department of Earth Science, University of Southern California, Los Angeles, California, USA, United States; University of Illinois at Chicago

**Keywords:** *Geobacter*, cytochromes, electroactive biofilm, nanowire, pili

## Abstract

Conductive nanowires are thought to contribute to long-range electron transfer (LET) in Geobacter sulfurreducens anode biofilms. Three types of nanowires have been identified: pili, OmcS, and OmcZ. Previous studies highlighted their conductive function in anode biofilms, yet a structural function also has to be considered. We present here a comprehensive analysis of the function of nanowires in LET by inhibiting the expression of each nanowire. Meanwhile, flagella with poor conductivity were expressed to recover the structural function but not the conductive function of nanowires in the corresponding nanowire mutant strain. The results demonstrated that pili played a structural but not a conductive function in supporting biofilm formation. In contrast, the OmcS nanowire played a conductive but not a structural function in facilitating electron transfer in the biofilm. The OmcZ nanowire played both a structural and a conductive function to contribute to current generation. Expression of the poorly conductive flagellum was shown to enhance biofilm formation, subsequently increasing current generation. These data support a model in which multiheme cytochromes facilitate long-distance electron transfer in G. sulfurreducens biofilms. Our findings also suggest that the formation of a thicker biofilm, which contributed to a higher current generation by G. sulfurreducens, was confined by the biofilm formation deficiency, and this has applications in microbial electrochemical systems.

## INTRODUCTION

Electroactive bacteria are well known to be able to use the anode as an electron acceptor to generate current via a process called extracellular electron transfer (EET) (1–5). EET not only lays the foundation of microbial fuel cells but also contributes to the operation of other bioelectrochemical processes, such as biosensing, microbial electrosynthesis, and bioelectrofermentation (2, 6, 7). However, EET is generally recognized as a deficient process that contributes to the low working efficiency in bioelectrochemical systems and thereby limits their applications. Understanding the mechanism of EET will guide endeavors to solve this deficiency. Geobacter sulfurreducens is one of the best-studied electroactive bacteria (8, 9). It forms a thick (up to 130 μm) biofilm on the anode that has generated the highest currents among reported electroactive bacteria. While cells on the anode surface can reduce it by direct contact, cells in the biofilm at a distance from the anode must have the ability to reduce the anode via long-range electron transport (LET). Accordingly, cytochromes and conductive nanowires have previously been hypothesized to facilitate LET (10, 11).

Two models of LET have been suggested. The multistep electron hopping model proposes that electrons are transferred by hopping between adjacent cytochromes in the biofilm (12–14). In contrast, the conductive pilus hypothesis suggests that G. sulfurreducens expresses conductive pili with metallic-like conductivity that transfer electrons directly to facilitate LET in the biofilm (11). Despite these competing models, it is generally recognized that cytochromes are involved in LET. For example, in both models, cytochrome OmcZ was shown to be necessary for efficient current generation (12, 15–18). In contrast, the function of the pili remains enigmatic, and even the identity and structural information of the pili are controversial (19–21). Primary attempts to detect pili in G. sulfurreducens biofilms by atomic force microscopy or cryo-electron microscopy have not identified pilus-like filaments but have identified two cytochrome nanowires: the OmcS and OmcZ nanowires (16, 22, 23). It has also been reported that both of these nanowires are conductive (16, 23). In particular, the OmcZ nanowire was shown to have a much higher conductivity than the OmcS nanowire (16). Furthermore, mutational analysis demonstrated that deletion of *omcZ* severely inhibited the current generation of G. sulfurreducens, while deletion of *omcS* only slightly inhibited the current generation (24). Recently, the expression of pili was directly verified by immunogold labeling against peptide-tagged pilin (25). It has even been suggested that pili are more abundant than OmcS nanowires in G. sulfurreducens cells growing with soluble electron acceptors (26). In addition, it was reported that inhibiting the expression of pili impaired current generation (27, 28). Therefore, it could be concluded that all three conductive nanowires are expressed in the anode biofilm and contribute to the current generation of G. sulfurreducens.

Biofilm formation on the anode is necessary for current generation by G. sulfurreducens since the bacterium cannot secrete a mediator to facilitate anode reduction (8, 29–31). Therefore, the formation of a thicker biofilm usually contributes to higher current generation (27). In particular, the formation of an electroactive and conductive G. sulfurreducens anode biofilm is impacted by several factors (32–34). In addition to the common limiting factors that also exist during the formation of other biofilms, such as mass transfer limitation, the formation of the anode biofilm was affected by the longitudinal redox gradient across the biofilm (10). Specifically, in a well-mixed reactor, redox gradient limitation prevailed. Interestingly, a recent study demonstrated that the expression of poorly conductive flagella was able to contribute to the formation of a thicker anode biofilm, most likely by breaking the redox gradient limitation (13), highlighting the structural function of nanofilaments in anode biofilm formation. Actually, the structural function of conductive pili in biofilm formation has been suggested but has not been verified (35).

**FIG 1:**
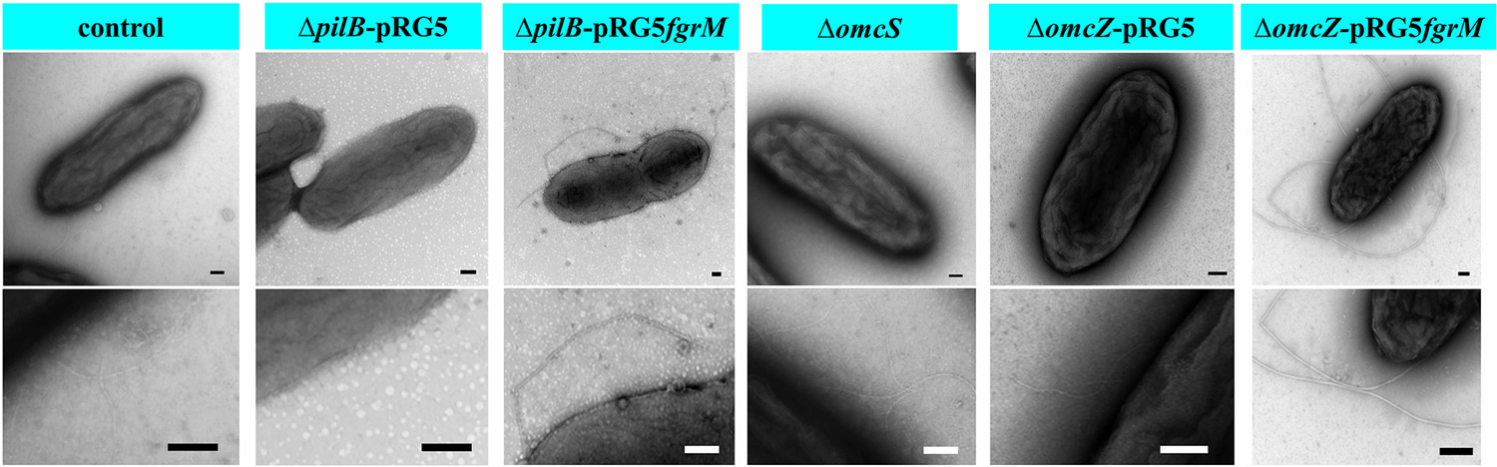
Representative transmission electron micrographs of G. sulfurreducens strains. The control strain was the wild-type strain G. sulfurreducens PCA carrying the empty vector pRG5. The G. sulfurreducens Δ*pilB*-pRG5 strain is deficient in pilus expression because of the deletion of the pilin assembly protein PilB, and it carries plasmid pRG5. The G. sulfurreducens Δ*pilB-*pRG5fgrM strain is the G. sulfurreducens *pilB*-deficient strain carrying the *fgrM* gene in *trans*. The G. sulfurreducens Δ*omcS* strain is deficient in the expression of OmcS nanowires, and strain G. sulfurreducens Δ*omcZ-*pRG5 is deficient in the expression of OmcZ nanowires and carries the plasmid pRG5. The G. sulfurreducens Δ*omcZ-fgrM* strain is a G. sulfurreducens *omcZ*-deficient strain carrying the *fgrM* gene in *trans*. Shown are representatives of at least five images for each strain. The lower panels show magnified views. Scale bar, 100 nm.

In this study, we examined all three nanowires in the anode biofilm of G. sulfurreducens with the goal of defining both their structural and conductive contributions. The encoding genes were deleted (Fig. 1) to inhibit the expression of specific nanowires. Specially, to study the structural contribution in the absence of conductivity, a poorly conductive flagellum was expressed in relevant nanowire mutants to restore structural function (Fig. 1). Accordingly, both current generation and biofilm formation in these strains were tested. The results demonstrated a structural function for pili and the OmcZ nanowire in anode biofilm formation and indicated a conductive function of OmcZ and OmcS nanowires in LET. These results are consistent with cytochrome-mediated electron transfer in G. sulfurreducens anode biofilm, highlight the structural functions of nanowires in contributing to anode biofilm formation and current generation, and provide potential pathways for increasing the efficiency of bioelectrochemical systems.

## RESULTS

### Pili play structural function in anode biofilm.

**FIG 2:**
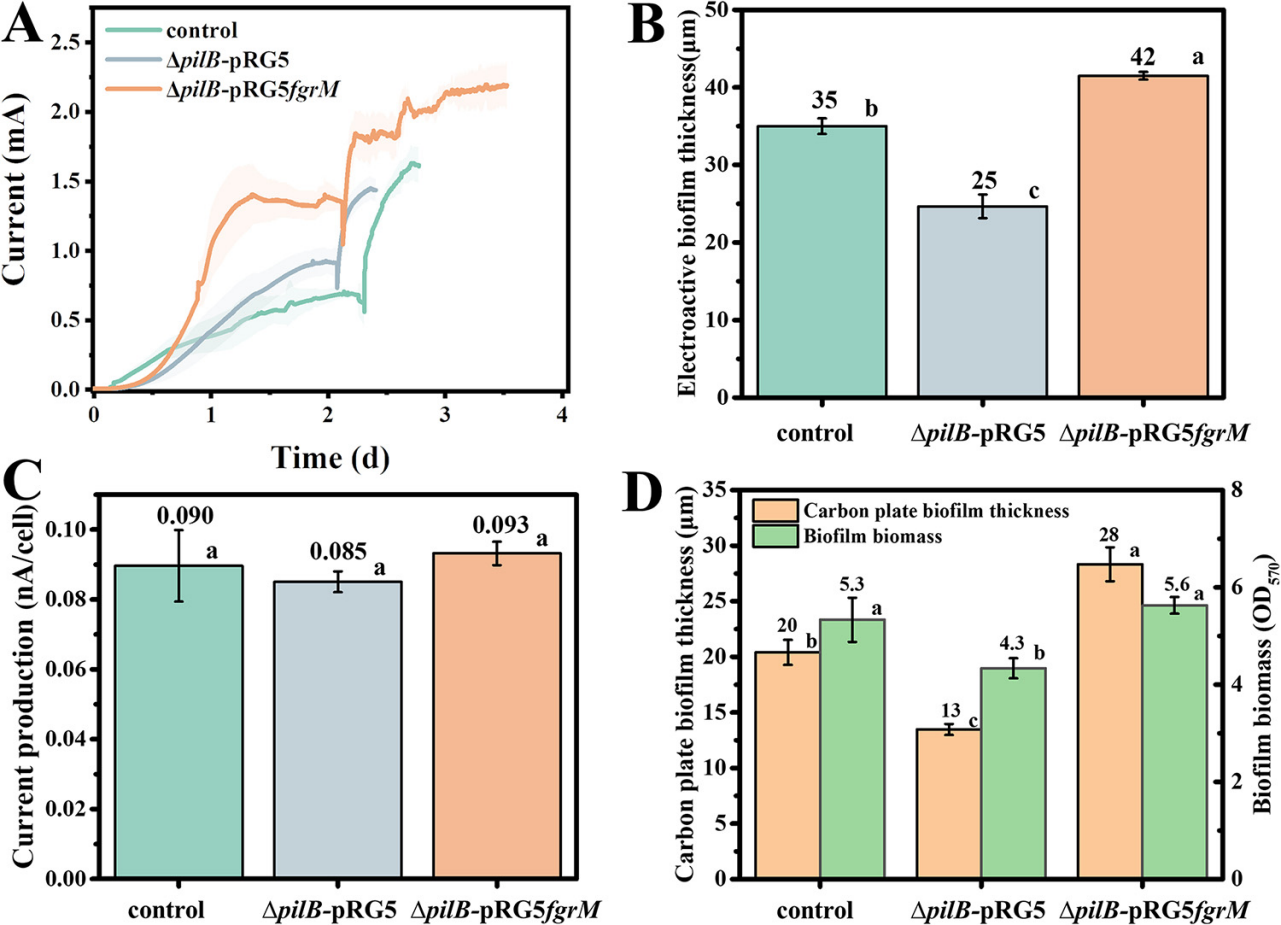
Current generation and biofilm formation of pili-deficient strains. (A) Averaged current generation of the control, G. sulfurreducens Δ*pilB-*pRG5, and G. sulfurreducens Δ*pilB-*pRG5*fgrM* strains. The shaded area represents one standard deviation. Three independent tests were performed for each strain. (B) Static calculation of anode biofilm thickness. Three biofilms were selected, and the thickness was measured at three different sites of each biofilm. (C) Normalized current generation per cell. (D) Static calculation of the biofilm thickness on a graphite carbon plate and of the biofilm biomass on a plastic surface. Three biofilms were measured. Columns with different letters are statistically different (LSD test, *P < *0.05).

G. sulfurreducens was previously reported to express conductive pili when growing on the anode (30, 36). A mutant strain named G. sulfurreducens Δ*pilB*-pRG5 was constructed by deleting the gene encoding the pilus assembly protein PilB ATPases as previously reported (27, 28). This strain is deficient in pili expression since it cannot power pilus polymerization, but it has an intact extracellular cytochrome profile, for example, having OmcZ and OmcS, and comparable metabolism (ferric citrate reduction) as in the control strain (28) (see Fig. S1). However, as indicated in Fig. 2A, strain Δ*pilB-*pRG5 produced a lower current of 1.44 ± 0.02 mA than that of the control strain (1.63 ± 0.11 mA). In addition, strain Δ*pilB-*pRG5 formed a thinner anode biofilm (ca. 25 μm) than the control strain (ca. 35 μm) (Fig. 2B; see also Fig. S2). Previous studies have indicated a conductive function of pili in facilitating electron transfer in anode biofilm (11, 27, 36). However, the calculated electron generation per cell was comparable between those two strains (0.090 ± 0.010 nA and 0.085 ± 0.003 nA for the control and G. sulfurreducens Δ*pilB-*pRG5 strains, respectively) (Fig. 2C). Furthermore, the calculated conductivity of the biofilm was also comparable between the two strains (1.06 μS cm^−1^ and 1.03 μS cm^−1^ for the control and the G. sulfurreducens Δ*pilB-*pRG5 strain, respectively) (see Fig. S3). Therefore, the absence of pili did not affect the electron transfer in the anode biofilm. A previous study also indicated the structural function of the pili in G. sulfurreducens biofilm on a nonreducible surface growing with a soluble electron acceptor (35). Similarly, strain G. sulfurreducens Δ*pilB-*pRG5 formed a less-dense biofilm not only on an unpolarized graphite carbon plate (ca. 13 μm) (see Fig. S4) but also on a plastic surface (OD_570_ of 4.3) compared to the control strain (ca. 20 μm and an optical density at 570 nm [OD_570_] of 5.3).

10.1128/mbio.03822-21.2 FIG S1 Reduction of ferric citrate by G. sulfurreducens wild-type strain (WT), control strain, and mutant strains. The results are presented as the means and standard deviations from three independent cultures of each strain. Download FIG S1, PDF file, 0.3 MB. Copyright © 2022 Ye et al. 2022 Ye et al. https://creativecommons.org/licenses/by/4.0/ This content is distributed under the terms of the Creative Commons Attribution 4.0 International license.

10.1128/mbio.03822-21.3 FIG S2 Representative confocal laser scanning microscopy images of anode biofilms from different G. sulfurreducens strains. Download FIG S2, PDF file, 0.7 MB. Copyright © 2022 Ye et al. 2022 Ye et al. https://creativecommons.org/licenses/by/4.0/ This content is distributed under the terms of the Creative Commons Attribution 4.0 International license.

10.1128/mbio.03822-21.4 FIG S3 Calculated conductivity versus gate potential response of G. sulfurreducens strains (A) control and (B) Δ*pilB*-pRG5 biofilms. The electrochemical gating measurements were directly performed on interdigitated microelectrode arrays (IDAs) using a biopotentiostat (CHI760E; CH Instruments, Inc.). The IDAs had a double-band configuration consisting of 130 antiparallel gold rectangular microelectrodes that had dimensions 2 mm long × 10 μm wide × 90 nm thick and were set apart by 5-μm-wide gaps. Potentials with a fixed offset bias of 10 mV (*V_SD_*) were applied on IDAs and scanned between −0.6 and 0.3 V with a slow rate of 1 mV s^−1^ simultaneously. The same measurement was also performed with an offset bias of 0 mV to calculate the total background current, which was subtracted from the current measured at a bias of 10 mV, generating the source and drain current at the individual gate potential (*E_G_*). The conductivity (σ) was calculated according to the following equation: σ = *I*_SD_/(*S*×*V*_SD_), scaling factor = 1/(*S*×*V*_SD_) = 4.9 cm^−1^ V^−1^. Download FIG S3, PDF file, 0.4 MB. Copyright © 2022 Ye et al. 2022 Ye et al. https://creativecommons.org/licenses/by/4.0/ This content is distributed under the terms of the Creative Commons Attribution 4.0 International license.

10.1128/mbio.03822-21.5 FIG S4 Representative confocal laser scanning microscopy images of biofilms from different G. sulfurreducens strains growing on graphite plates. Download FIG S4, PDF file, 0.8 MB. Copyright © 2022 Ye et al. 2022 Ye et al. https://creativecommons.org/licenses/by/4.0/ This content is distributed under the terms of the Creative Commons Attribution 4.0 International license.

G. sulfurreducens strain PCA could not express a flagellum because of the absence of an RpoN-dependent enhancer-binding protein FgrM essential for the synthesis of a flagellum (13, 37). To identify the possible structural function of pili in the anode biofilm, flagellum expression was induced in strain Δ*pilB-*pRG5 by expressing the gene encoding FgrM in *trans*, generating strain G. sulfurreducens Δ*pilB-*pRG5*fgrM*, in an effort to compensate for the possible deficiency in structural function but not conductivity, since the flagellum is poorly conductive (11) and plays a structural function in the G. sulfurreducens strain KN400 anode biofilm to support the formation of a thick biofilm (13). Furthermore, the expression of the flagellum did not affect the extracellular cytochrome profile of G. sulfurreducens (13). Another option is to reduce or inhibit the conductivity of native pili but keep the structure intact by replacing key aromatic amino acids with alanine (27, 38). However, this is usually incurred at altering the extracellular cytochrome profile since the pili belong to the type II secretion system (27, 39, 40). In G. sulfurreducens strain Δ*pilB-*pRG5*fgrM*, the expression of flagellum recovered the biofilm formation deficiency of strain Δ*pilB-*pRG5, even resulting in the formation of a thicker biofilm not only on the anode (ca. 42 μm) (Fig. 2B; see also Fig. S2) but also on the unpolarized graphite carbon plate (ca. 28 μm) (see Fig. S4) and the plastic surface (OD_570_ of 5.6) (Fig. 2D). Moreover, flagellum expression recovered the current generation of strain G. sulfurreducens Δ*pilB-*pRG5 to generate a higher current of 2.20 ± 0.16 mA than the control strain (Fig. 2A). However, flagellum expression had no effect on current generation (electron transfer) per cell in the anode biofilm (Fig. 2C), showing no conductivity contribution. Considering these data, the structural function of pili in the anode biofilm could be concluded. Moreover, there is a shorter lag time for current generation in strain G. sulfurreducens Δ*pilB-*pRG5fg*rM* (Fig. 2A), possibly due to motility promoting the primary colonization of bacterial cells on the anode (13, 41).

### OmcS nanowires contribute to electron transfer in anode biofilm.

**FIG 3:**
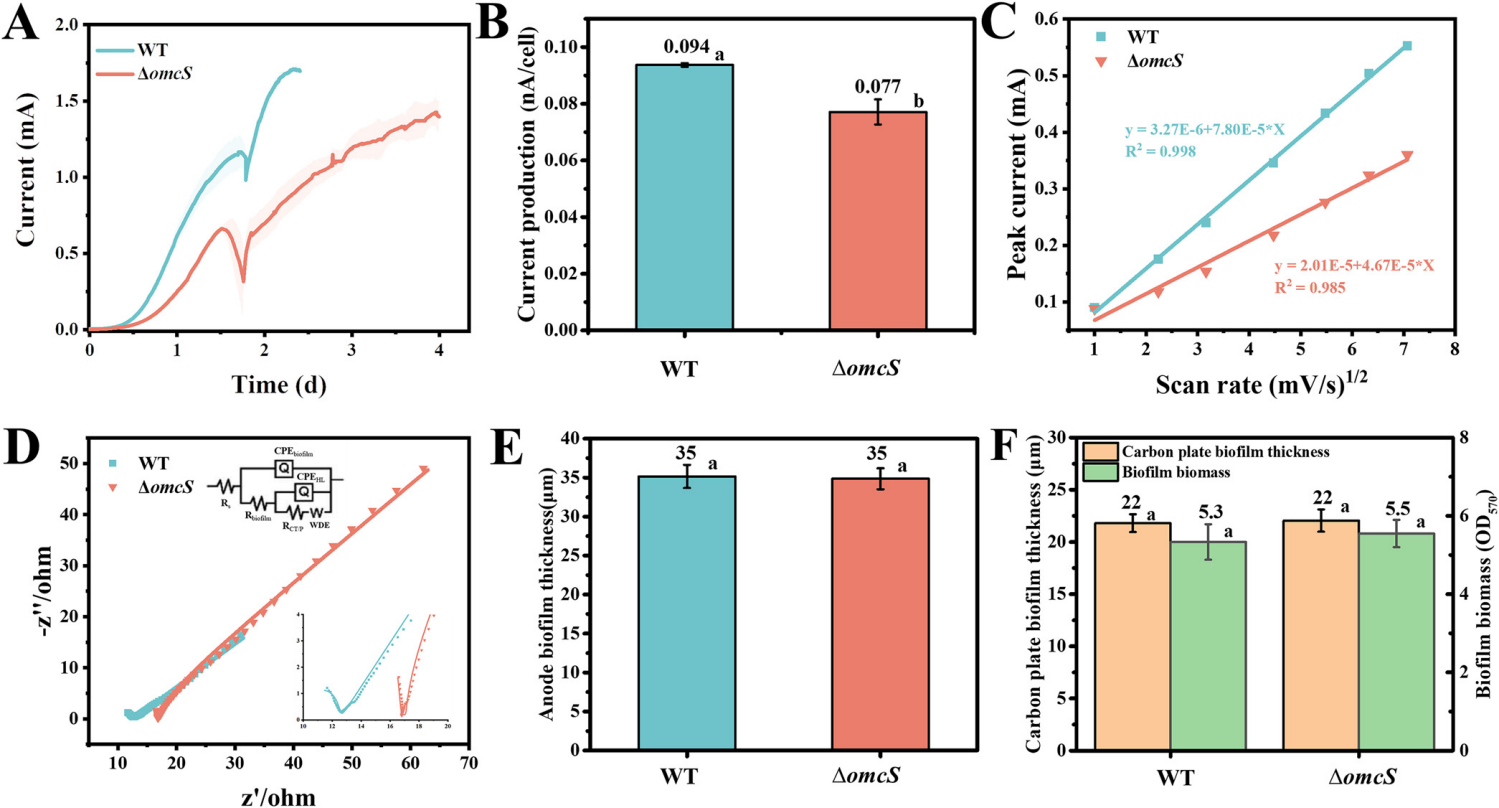
Current generation, biofilm formation, and electrochemical kinetic characterization of the OmcS mutant. (A) Averaged current generation of the G. sulfurreducens wild-type strain (WT) and strain Δ*omcS*. The shaded area represents one standard deviation. Three independent tests were performed for each strain. (B) Normalized current generation per cell. (C) Linear dependence of baseline-subtracted oxidation peak current height in cyclic voltammogram (see Fig. S5) with the square root of the scan rate. A higher slope indicates lower electron transfer resistance in the biofilm. (D) Nyquist plot with inset showing the equivalent circuit and the magnification of the high-frequency region. The equivalent circuit was adopted from a previous model (57) and consisted of resistance due to solution (Rs), two time constants, namely, double-layer capacitance of biofilm (CPE_biofilm_) and Helmholtz layer capacitance (CPE_HL_), ascribed to the biofilm and the anode, respectively, which were linked in parallel to their corresponding resistance of biofilm (R_biofilm_) and charge transfer or polarization (R_CT/P_), followed by Warburg’s diffusion element (W). (E) Static calculation of the anode biofilm thickness. Three biofilms were selected and the thickness was measured at three different sites of each biofilm. (F) Static calculation of the biofilm thickness on the graphite carbon plate and the biofilm biomass on the plastic surface. Three biofilms were measured. Columns with different letters are statistically different (LSD test, *P < *0.05).

Previous studies have demonstrated that cytochrome OmcS could polymerize to form a conductive nanowire and was shown to be highly expressed in anode biofilms (22, 23). To inhibit the expression of the OmcS nanowire, the *omcS* gene was deleted, generating strain Δ*omcS*. The deletion of *omcS* inhibited the expression and production of OmcS (42) but did not affect the metabolism (reduction of ferric citrate) of the cell (see Fig. S1). As shown in Fig. 3A, strain G. sulfurreducens Δ*omcS* generated a lower current of 1.43 ± 0.10 mA than the wild-type strain (1.70 ± 0.02 mA), which was similar to results seen in a previous study (24). In particular, the calculated current generation per cell of the Δ*omcS* strain (0.077 ± 0.004 nA) was lower than that of the wild-type strain (0.094 ± 0.001 nA) (Fig. 3B), indicating that the absence of OmcS impaired electron transfer in the anode biofilm.

10.1128/mbio.03822-21.6 FIG S5 Cyclic voltammograms at increased scan rates under nonturnover conditions for G. sulfurreducens WT strain and strain Δ*omcS* biofilms. Cyclic voltammetry was performed *in situ* under nonturnover conditions by scanning the biofilm electrode from 0.3 to −0.6 V. Download FIG S5, PDF file, 0.4 MB. Copyright © 2022 Ye et al. 2022 Ye et al. https://creativecommons.org/licenses/by/4.0/ This content is distributed under the terms of the Creative Commons Attribution 4.0 International license.

Further electrochemical kinetic analyses (Fig. 3C and D; see also Fig. S5) also demonstrated a higher electron transfer resistance in the Δ*omcS* anode biofilm with a calculated *R*_biofilm_ of 9.6 Ω compared to the *R*_biofilm_ of 3.2 Ω in the WT anode biofilm. Therefore, the OmcS nanowire could facilitate electron transfer in the anode biofilm. In contrast, the biofilm on the anode (Fig. 3E; see also Fig. S2), the unpolarized graphite carbon plate (Fig. 3F; see also Fig. S2) or on the plastic surface (Fig. 3F) displayed a comparable thickness between those two strains. Therefore, the OmcS nanowire did not appear to play a significant structural role in the G. sulfurreducens biofilm.

### OmcZ nanowires have both structural functions and conductive functions in anode biofilm.

**FIG 4:**
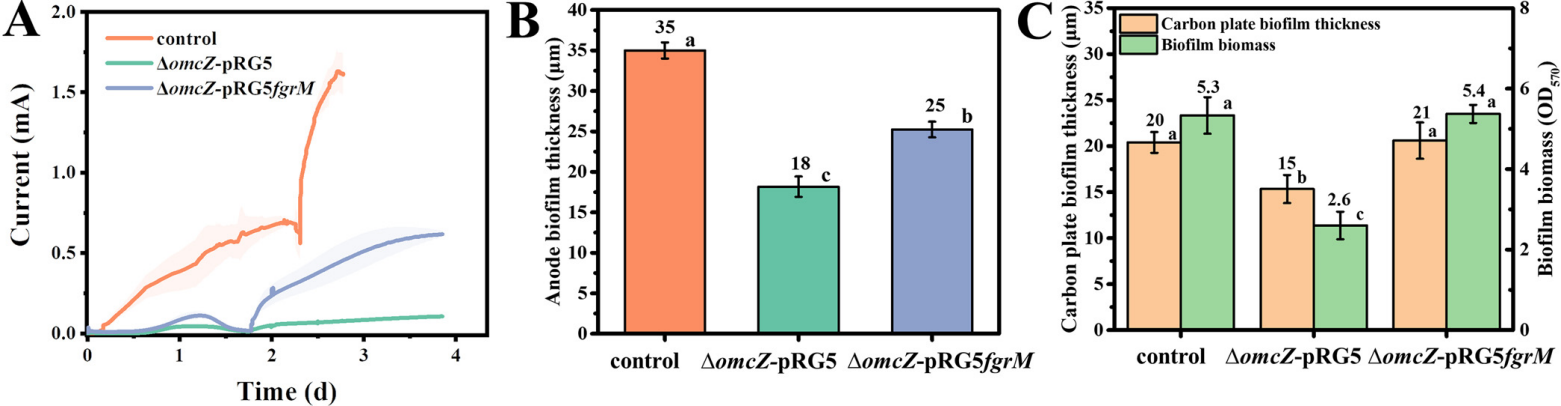
Current generation and biofilm formation of an OmcZ mutant. (A) Average current generation of the control, G. sulfurreducens Δ*omcZ-*pRG5, and G. sulfurreducens Δ*omcZ-*pRG5*fgrM* strains. The shaded area represents one standard deviation. Three independent tests were performed for each strain. (B) Static calculation of anode biofilm thickness. Three biofilms were selected, and the thickness was measured at three different sites of each biofilm. (C) Static calculation of the biofilm thickness on the graphite carbon plate and the biofilm biomass on the plastic surface. Three biofilms were measured. Columns with different letters are statistically different (LSD test, *P < *0.05).

It was previously reported that the OmcZ cytochrome was also highly expressed in the G. sulfurreducens anode biofilm (43) and performed vital functions in current generation (15, 16). A recent report indicated that OmcZ cytochromes polymerize to form conductive nanowires in anode biofilm (16). To further inhibit OmcZ nanowire expression, the *omcZ gene* was deleted (24), generating the G. sulfurreducens Δ*omcZ-*pRG5 strain. As indicated in Fig. S1, the deletion of *omcZ* did not affect the ferric citrate reduction of G. sulfurreducens. However, current generation was severely inhibited with a low current of 0.12 ± 0.01 mA (Fig. 4A). This is consistent with previous studies showing the vital function of OmcZ in current generation (12, 16). Accordingly, strain G. sulfurreducens Δ*omcZ-*pRG5 generated a much thinner scattered anode biofilm (see Fig. S2) with a maximum thickness of ca. 18 μm (Fig. 4B). Unexpectedly, strain G. sulfurreducens Δ*omcZ-*pRG5 also showed impaired biofilm formation on the unpolarized graphite carbon plate (Fig. 4C; see also Fig. S4) and on the plastic surface (Fig. 4C), indicating a previously unsuspected structural role in biofilm formation.

Flagellum expression was also induced in the G. sulfurreducens *ΔomcZ* strain by expressing FgrM in *trans*, generating strain G. sulfurreducens Δ*omcZ-*pRG5*fgrM*. As indicated in Fig. 4C, the expression of the flagellum could compensate for the structural deficiency of the OmcZ nanowire in supporting biofilm formation both on an unpolarized graphite carbon plate and on a plastic surface to achieve a thickness comparable to that of the wild-type strain (Fig. 4C; see also Fig. S4). However, flagellum expression only partially recovered anode biofilm formation to a thickness of 25 μm (Fig. 4B; see also Fig. S2). Accordingly, the current generation was partially recovered with a maximum of 0.63 ± 0.01 mA (Fig. 4A). Therefore, the OmcZ nanowire should also facilitate electron transfer in the anode biofilm, displaying a conductive function. Electrochemical kinetic analyses showed increased electron transfer resistance in the absence of OmcZ (see Fig. S6).

10.1128/mbio.03822-21.7 FIG S6 Linear dependence of baseline-subtracted oxidation peak current height in cyclic voltammogram with the square root of the scan rate. Download FIG S6, PDF file, 0.4 MB. Copyright © 2022 Ye et al. 2022 Ye et al. https://creativecommons.org/licenses/by/4.0/ This content is distributed under the terms of the Creative Commons Attribution 4.0 International license.

### OmcZ extracellular cytochromes mainly contribute to electron transfer in the anode biofilm.

**FIG 5:**
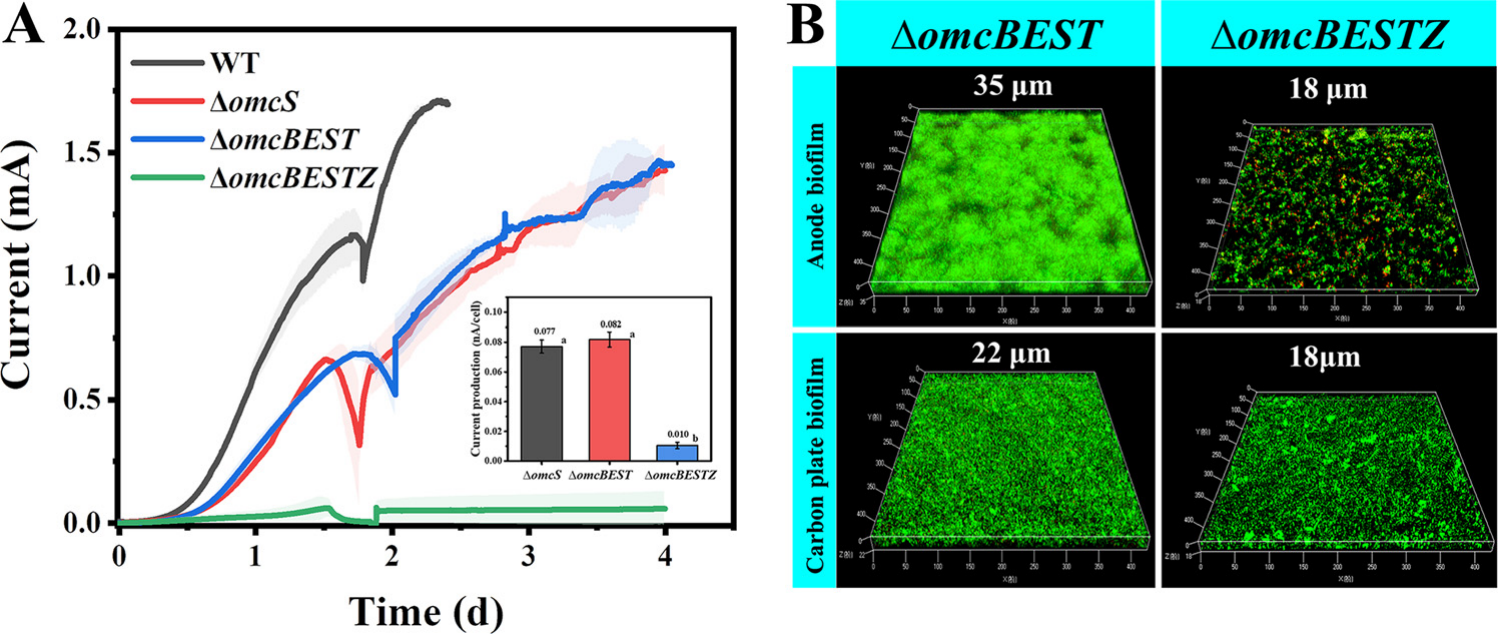
Current generation and biofilm formation by extracellular cytochrome mutants. (A) The current generation of the G. sulfurreducens wild-type (WT) strain, the G. sulfurreducens Δ*omcS* strain, the G. sulfurreducens Δ*omcBEST* strain (which is deficient in the expression of quadruple extracellular cytochromes, OmcB, OmcE, OmcS, and OmcT), and the G. sulfurreducens Δ*omcBESTZ* strain, which is deficient in the expression of quintuple extracellular cytochromes of OmcB, OmcE, OmcS, OmcT, and OmcZ. The shaded area represents the standard deviation. Three independent tests were performed for each strain. The inset shows the calculated current generation of a single cell. Columns with different letters are statistically different (LSD test, *P < *0.05). (B) Representative images of the anode biofilm and of biofilm growing on the graphite carbon plate from the G. sulfurreducens Δ*omcBEST* and G. sulfurreducens Δ*omcBESTZ* strains.

In the anode biofilm, G. sulfurreducens was also shown to secrete other cytochromes, including OmcB, OmcE, and OmcT (42, 44). Previous studies have indicated the importance of cytochromes in facilitating electron transfer in anode biofilms (10, 12). To identify the function of those cytochromes in the anode biofilm, the quadruple and quintuple G. sulfurreducens Δ*omcBEST* and G. sulfurreducens Δ*omcBESTZ* cytochrome mutant strains, respectively, were constructed. As indicated in Fig. 5A, the G. sulfurreducens *ΔomcBEST* strain generated a current (1.47 ± 0.01 mA) much the same as the G. sulfurreducens *ΔomcS* strain. Furthermore, when comparing these two strains, the biofilm thicknesses (Fig. 5B) and the calculated current generation of single cells (Fig. 5A) were the same. Therefore, the cytochromes OmcB, OmcE, OmcS, and OmcT did not contribute greatly to biofilm formation and electron transfer in the anode biofilm under the conditions tested. In contrast, the additional deletion of *omcZ* in the G. sulfurreducens Δ*omcBEST* strain (strain Δ*omcBESTZ*) inhibited both current generation and biofilm formation (Fig. 5A), providing additional evidence highlighting the importance of OmcZ in the anode biofilm.

## DISCUSSION

**FIG 6:**
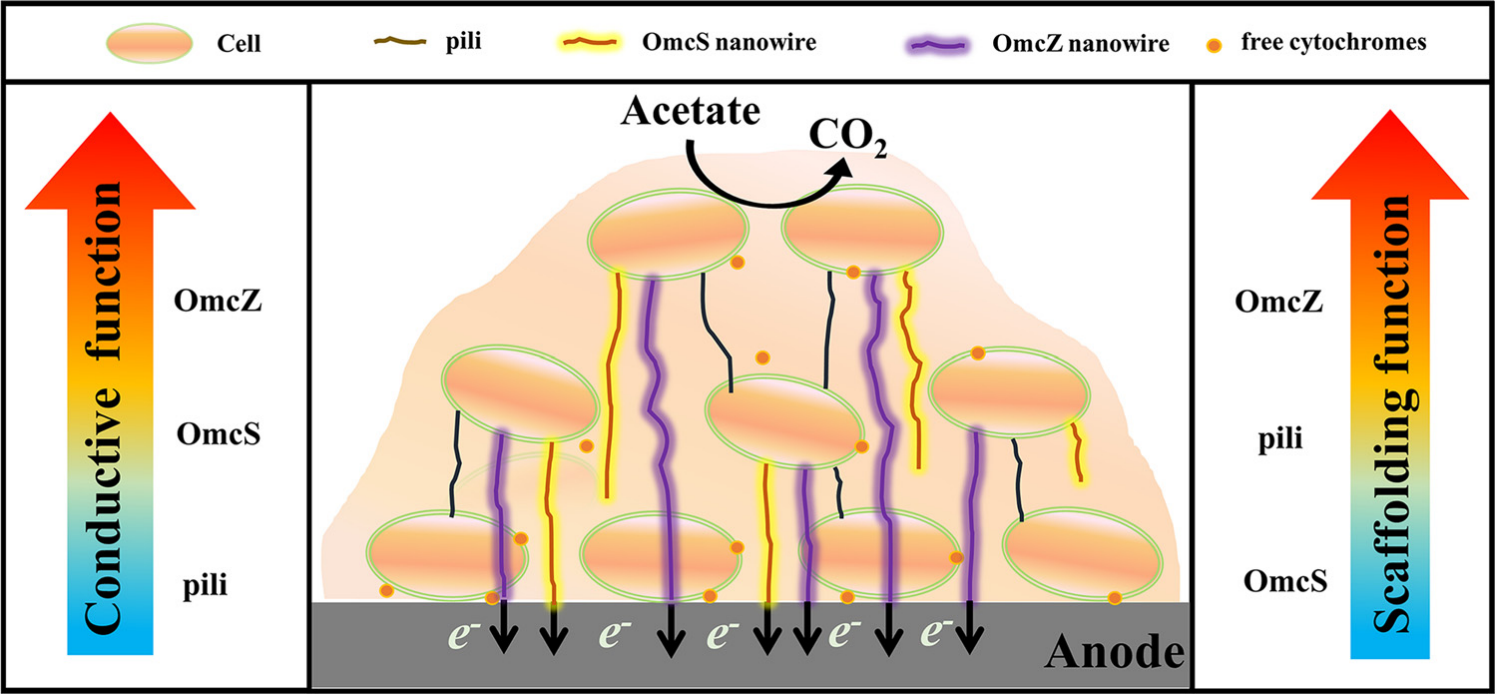
Model of nanowires participating in electron transfer in the G. sulfurreducens anode biofilm.

Our results have led to the formation of a working model (Fig. 6) of nanowires in G. sulfurreducens anode biofilm: (i) the pili play only a structural role supporting the formation of a thick anode biofilm; (ii) OmcS plays only a conductive role in facilitating LET; and (iii) the OmcZ nanowire plays both conductive and structural roles, contributing to both biofilm formation and primary current generation. Specifically, it was observed that the biofilm of strain Δ*pilB-*pRG5 on either the graphite carbon plate or plastic surface was thicker than the biofilm of strain Δ*omcZ*, suggesting that pili played a minor structural role in biofilm formation. The reason might be that G. sulfurreducens did not express pili abundantly compared to OmcZ nanowires (16). Similarly, it was observed that strain Δ*omcZ*-pRG5*fgrM* generated a much lower current than the Δ*omcS* strain, suggesting a higher electron transfer contribution of OmcZ nanowires compared to OmcS nanowires in the anode biofilm. This is understandable since OmcZ nanowires have a much higher conductivity than OmcS nanowires (16). Moreover, the results highlighted the importance of cytochromes in the anode biofilm and provided evidence to support a model in which cytochromes mediate electron transfer in the electroactive biofilm.

The finding that the pili played only a structural role in the anode biofilm is consistent with a previous study showing that increasing the conductivity of pili did not affect the anode biofilm thickness and current generation (45). Nevertheless, this result contradicts previous studies suggesting that pili provide an electron transfer pathway to facilitate electron transfer (11, 37) directly. In those studies, the pilus-encoding gene *pilA* was directly deleted to inhibit the expression of conductive pili, and the resulting deletion mutant was deficient in both current generation and biofilm formation. However, as indicated in recent studies, the deletion of *pilA* also blocked the secretion of some key extracellular cytochromes, such as OmcS and OmcZ (28, 39). With our results showing that OmcZ is necessary in both current generation and biofilm formation, the phenotype of the *pilA* mutant should be attributed to the absence of those key cytochromes in the extracellular matrix. In contrast, the deletion of the gene encoding the pilin assembly protein PilB ATPase was shown to inhibit the expression of pili but did not affect the profile of extracellular cytochromes (28). Notably, a recent study suggested that in the absence of the PilB ATPase, G. sulfurreducens could still express pili (46). However, this conclusion should not be applicable to our Δ*pilB* mutant since in that study, the only direct evidence of immunogold labeling came from the study of G. sulfurreducens strain KN400 (4), which has a significantly different genetic background than strain PCA used in our study (47).

The same *pilB* deletion mutant as in our study was also constructed in a previous paper to study the function of pili (27). In that report, the *pilB* mutant also displayed both deficient current generation and biofilm formation. Particularly, to exclude the structural contribution of pili to anode biofilm formation and then current generation, the conductivity of pili was inhibited after replacing tyrosines in the pilus electron transfer pathway with alanines to generate G. sulfurreducens strain Tyr3. The results demonstrated that strain Tyr3 was impaired in the current generation but not in biofilm formation on a plastic surface (27). Therefore, the conductivity function of pili was concluded, and it was thus further concluded that a high conductivity of the pili was necessary for the formation of a thick anode biofilm (27). This is in contrast to our results showing that nonconductive flagella were able to restore the deficiency of pili to grow an even thicker biofilm and generate a higher current and that pili had only a structural function. We credit the discrepancy to the altered extracellular cytochrome profiles in strain Tyr3 compared to the wild-type strain (27), which might impair electron transfer in anode biofilm.

The multistep electron hopping model also supported the notion of a structural role of pili in the anode biofilm (12). In that model, the pili were predicted to act as scaffolds for the binding of cytochromes, such as OmcS. This model contrasts with our model showing a structural role of pili in anode biofilm formation and requires revision since recent reports demonstrated that cytochromes such as OmcS and OmcZ could form nanowires by themselves (16, 23). Notably, a very recent study suggested a model in which pili were composed of PilA-N and PilA-C heterodimers and were only expressed in the periplasm akin to type II secretion pseudopili facilitating cytochrome secretion (39). Such a model seems unlikely when considering that evidence of the secretion of PilA-N has been verified and well documented by different research groups (20, 48, 49); that PilA-C has been shown to form a trimer in the inner membrane acting as a pilin chaperone (50); that the measured diameter of pili is ∼3 nm, which is much thinner than the diameter (∼6.5 nm) of the proposed pseudopili (26, 39); and that the G. sulfurreducens COMB strain could express thick pili composed of fused PilA-N and PilA-C by fusing *pilA-N* and *pilA-C* to facilitate electron transfer in anode biofilms (20).

The finding that the expression of nonconductive flagella could increase anode biofilm formation motivates rethinking of the factors determining the thickness of the anode biofilm. Previous studies have indicated that electron donor transport limitations, proton accumulation and redox gradient dissipation across the biofilm determine the thickness of anode biofilm (33, 34, 51, 52). The mass transport limitation should not be dominant in our system since the electrolyte was well stirred and had a high buffer capacity and since the biofilm thickness was below the threshold incurring mass transport limitations (27). It was suggested that the redox gradient across the biofilm drove electron flow toward the anode (10). Specially, the redox potential decreased progressively with distance from the anode (53, 54). Therefore, the reduced species accumulated at the outer layer of the biofilm, forming a redox gradient too low to drive electron transfer, and cells in the outer layer were kept at a minimum metabolism too low to grow a thicker biofilm (32, 52). It has further been suggested that the expression of conductive pili could overcome the redox gradient limitation to form a thick biofilm by transferring electrons to underlying oxidized cytochromes or to the anode (27). However, a previous study (13) and our results demonstrated that the expression of nonconductive flagella was also able to increase the anode biofilm thickness. Furthermore, we showed that the deletion of conductive OmcS nanowires increased electron transfer resistance but did not impair biofilm formation. Previous studies showed that neither increasing nor inhibiting the conductivity of pili affected the thickness of the anode biofilm (38, 45). Therefore, we speculate that redox gradient limitation does not primarily determine the biofilm thickness but that the deficiency of biofilm formation confines the thickness of the wild-type G. sulfurreducens anode biofilm.

It is feasible to study the function of OmcS and OmcZ by targeted deletion since a deletion did not affect the expression of other cytochromes and structural components (see Fig. S7), such as pili and exopolysaccharides (55). Our study provides further evidence to support the model in which cytochromes facilitate electron transfer in anode biofilms. Previous studies have demonstrated that OmcS nanowires are steadily expressed and distributed evenly in anode biofilms (19, 23). In contrast, OmcZ nanowires were shown to be expressed mainly at the biofilm-anode interface since their expression could be stimulated by an electric field (16). They are thus expected to act as electrochemical gates assisting interface electron transfer (43). Even though our study demonstrated a structural role of OmcZ in the anode biofilm, the finding that the expression of flagella could partially restore the current generation and anode biofilm formation of an OmcZ mutant challenged the presumption of an electrochemical gate role of OmcZ and suggested that OmcZ nanowires were responsible for the construction of the major portion of the electron transfer pathway at the surface of the anode.

10.1128/mbio.03822-21.8 FIG S7 Fold changes in the relative expression profiles of the GSU1501 (A), *pilA* (B), *omcS* (C), and *omcZ* (D) genes in G. sulfurreducens wild-type strain (WT), the control strain, and Δ*pilB-*pRG5, Δ*pilB-*pRG5*fgrM*, Δ*omcS*, Δ*omcZ*-pRG5, Δ*omcZ-p*RG5*fgrM*, Δ*omcBEST*, and Δ*omcBESTZ* mutant strains, respectively. Mean values and standard deviations were obtained from three independent cultures. Statistically significant changes in gene expression were determined using a *t* test. The expression of each gene was relative quantified by qPCR normalization against the housekeeping gene *rpoD* (GSU3089). Download FIG S7, PDF file, 0.7 MB. Copyright © 2022 Ye et al. 2022 Ye et al. https://creativecommons.org/licenses/by/4.0/ This content is distributed under the terms of the Creative Commons Attribution 4.0 International license.

Here, we have demonstrated the feasibility of compensating for a structural deficiency by expressing flagella and suggested a decisive structural role of flagella in G. sulfurreducens biofilm. Flagella are usually recognized as the locomotive organelle responsible for chemotactic movements in cells. However, flagella have also been shown to be highly expressed in the anode biofilm of G. sulfurreducens strain KN400 and act as biofilm scaffolds accommodating more cells and cytochromes to contribute to higher current generation and thicker biofilm formation (13). Therefore, in our study, flagella were expressed in specific mutant strains to compensate for a possible structural deficiency. The structural role was tested by growing G. sulfurreducens strains on both a graphite carbon plate and a plastic surface with fumarate as an electron acceptor, as previously reported (35). In the absence of pili, the expression of flagella contributed to the formation of a thicker biofilm compared to the control strain, indicating that flagella had a stronger structural role than pili. Similarly, in the absence of OmcZ, the expression of flagella restored biofilm formation to a thickness as displayed by the control strain, indicating a strong structural role for the OmcZ nanowire comparable to flagella. Furthermore, the finding that the expression of flagella further increased the current generation may provide a strategy to increase the efficiency and biofilm thickness in bioelectrochemical systems.

## MATERIALS AND METHODS

### Bacterial strains and culture conditions.

All strains used in this study are listed in Table S1. G. sulfurreducens wild-type strain PCA (ATCC 51573) and the control strain were acquired from our laboratory culture collection. The control strain was G. sulfurreducens wild-type strain carrying plasmid pRG5, which was named G. sulfurreducens PCA-pRG5 in a previous study and was constructed by transferring the empty expression plasmid pRG5 into wild-type G. sulfurreducens PCA (13). Strains Δ*pilB-*pRG5 and Δ*pilB-*pRG5fgrM were constructed similarly by transferring plasmids pRG5 and pRG5-*fgrM*, respectively, in a *pilB*-deficient G. sulfurreducens strain (named strain GS-ΔpilB in a previous study [28]). Three fragments were prepared to construct the G. sulfurreducens Δ*omcS* strain: the primer pairs omcSupF/omcSupR and omcSdnF/omcSdnR were used to amplify the sequences 500 bp upstream and 500 bp downstream, respectively, of GSU2504, using G. sulfurreducens genomic DNA as a template, and the primer pair gentF/gentR was used to amplify the gentamicin resistance cassette flanked by *loxP* sites from plasmid pCM351. These three fragments were connected with the linear pUC19 plasmid using an In-Fusion HD cloning kit as previously reported (28), generating plasmid pUC-*omcS*. This plasmid was linearized with ScaI and then electroporated into electrocompetent G. sulfurreducens cells. The G. sulfurreducens Δ*omcS* mutant strain was selected on NBAF agar plates containing gentamicin and verified by PCR. The *omcZ* mutant was constructed similarly. The pRG5 plasmid was further transferred into the *omcZ* mutant to construct strain G. sulfurreducens Δ*omcZ-*pRG5. Similarly, the pRG5-*fgrM* plasmid was transferred into the *omcZ* mutant to generate the G. sulfurreducens Δ*omcZ-*pRG5*fgrM* strain. To construct multiple cytochrome mutants, the genes encoding the cytochromes were mutated one by one following the same mutation procedure. Detailed steps can be found in Text S1. Since the same gentamicin selection marker was used for all mutants, the gentamicin cassette was deleted in the parental strains by transformation with the plasmid pCM158, which was able to express Cre recombinase as previously reported (28). Primer pairs used to construct the plasmids used for deletion and deletion verification are listed in Table S2. All strains were routinely cultured in NBAF medium containing 15 mM acetate as an electron donor and 40 mM fumarate as an electron acceptor under anaerobic conditions (80:20 N_2_:CO_2_) at 30°C as previously reported (56).

10.1128/mbio.03822-21.1 TEXT S1 Mutant construction. Download Text S1, PDF file, 0.08 MB. Copyright © 2022 Ye et al. 2022 Ye et al. https://creativecommons.org/licenses/by/4.0/ This content is distributed under the terms of the Creative Commons Attribution 4.0 International license.

10.1128/mbio.03822-21.9 TABLE S1 Bacterial strains and plasmids used in this study. Download Table S1, PDF file, 0.4 MB. Copyright © 2022 Ye et al. 2022 Ye et al. https://creativecommons.org/licenses/by/4.0/ This content is distributed under the terms of the Creative Commons Attribution 4.0 International license.

10.1128/mbio.03822-21.10 TABLE S2 Primers used for mutant construction and sequence. Download Table S2, PDF file, 0.3 MB. Copyright © 2022 Ye et al. 2022 Ye et al. https://creativecommons.org/licenses/by/4.0/ This content is distributed under the terms of the Creative Commons Attribution 4.0 International license.

### Three-electrode system construction and operation.

All electrochemical experiments were performed in a three-electrode system consisting of a dual-chambered H-shaped microbial fuel cell with the anode and the cathode chamber separated by a proton exchange membrane as previously described (13). The electrodes were made from a graphite carbon plate with dimensions of 30 × 20 × 3 mm and polished by a P1500 grit. The electrolyte was an anaerobic freshwater medium (FWNN) (13), with the addition of 15 mM acetate in the anode chamber as the sole electron donor for cell growth. To induce current generation, a constant voltage of +0.3 V (versus Ag/AgCl sat. KCl) was applied on the working electrode, and the current was recorded simultaneously by a potentiostat (CH Instrument, Inc., Shanghai, China). To prepare the inoculum, G. sulfurreducens growing in NBAF medium was collected by centrifugation (8,000 × *g*, 8 min) and washed twice with FWNN.

### Electrochemical analysis.

Cyclic voltammetry (CV) was performed under nonturnover conditions when the current declined to below 10^−6^ A. The voltammograms were recorded on a CHI660E (CH Instrument) by scanning potential from −0.6 V to 0.3 V with a gradient scanning rate. Electrochemical impedance spectroscopy was performed at open-circuit voltage with a perturbation amplitude of 5 mV. The frequency varied from 10,000 to 0.1 Hz. Nyquist plots and circle fitting software Zman were used to analyze the EIS data. Impedance spectra were fitted into the equivalent circuit model as in previous studies (57).

### Confocal laser scanning microscopy analysis.

The anode biofilm was grown in a three-electrode system. When the current reached the maximum, the anode was removed and rinsed with 0.9% NaCl to remove floating cells. To grow biofilms on graphite carbon plates with fumarate as an electron acceptor, the anode was unpolarized and immersed in FWNN medium supplied with 15 mM acetate and 40 mM fumarate. The biofilm was grown anaerobically at 30°C for 5 days. All biofilms were stained with Live/Dead stain (Live/Dead BacLight bacterial viability kit; Thermo Fisher Scientific, Waltham, MA) as previously reported (11). A confocal laser scanning microscope (Carl Zeiss, Jena, Germany) equipped with a 20× lens objective was used to obtain images.

### Biofilm formation assay.

An assay was performed in 96-well microtiter plates as previously described to measure biofilm formation on nonpolarized surfaces (58, 59). The bacterial strains were first grown in NBAF medium at 30°C until an OD_600_ of 0.4 was reached. Each well of the microtiter plate was filled with 250 μL of NBAF medium and inoculated with 10 μL of the cell culture. The uninoculated wells were used as the negative control. The well was drained and washed carefully with distilled water after incubation at 30°C for 60 h. The rest of the biofilm was then stained with 250 μL of 0.1% crystal violet for 30 min at room temperature, after which the well was drained and washed three times with distilled water. The bound crystal violet was solubilized with 250 μL of 92% ethanol. The amount of crystal violet was measured at OD_570_ with a multifunctional plate reader SpectraMax i3 analysis system (Multi-Mode Detection Platform, USA).

### Single-cell current production calculation.

Cells were quantified by quantitative PCR (qPCR) using the total DNA as the template. Briefly, a standard curve was prepared by cloning the GSU2751 gene into the pUCm-T vector (Sangon Biotech, China). The gene GSU2751 was selected because it is present as a single copy on the G. sulfurreducens genome and has been widely used for cell quantification in previous studies (28, 31). The whole biofilm was scraped off the anode when a maximum current was generated and then used directly for DNA extraction (FastDNA spin kit; MP Biomedicals, Irvine, CA) according to the manufacturer’s directions. Thereafter, the DNA was used as a template to perform qPCR with the primer pair qGSU2751f qGSU2751r (see Table S2). The gene GSU2751 copy number was calculated against the standard curve and used to reflect the cell number. The current generated by a single cell was calculated by dividing the maximum current by the number of cells in the anode biofilm.
