# Supplementary material for: Dissecting the Structural and Conductive Functions of Nanowires in Geobacter sulfurreducens Electroactive Biofilms
Source: mBio. 2022 Feb 15;13(1):e03822-21. doi: 10.1128/mbio.03822-21 (PMC8844916; doi:10.1128/mbio.03822-21)
Supplement: FIG S1 [file mbio.03822-21-sf001.pdf]

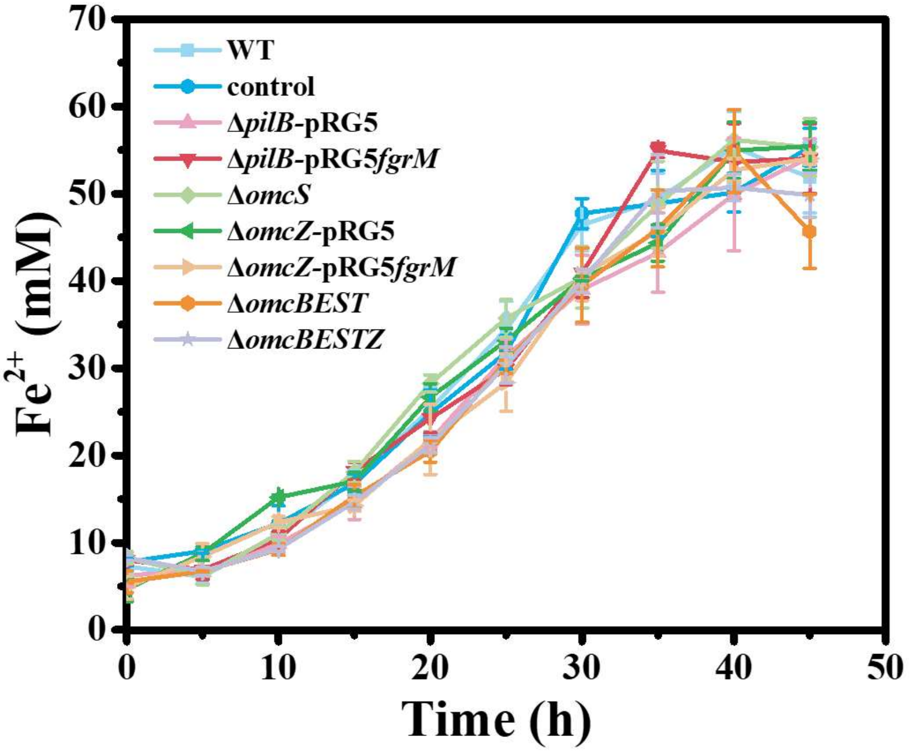

Figure S1. Reduction of ferric citrate by *G. sulfurreducens* wild-type strain (WT), control strain and mutant strains. The results are presented as the means and standard deviations from three independent cultures of each strain.
