# Supplementary material for: Dissecting the Structural and Conductive Functions of Nanowires in Geobacter sulfurreducens Electroactive Biofilms
Source: mBio. 2022 Feb 15;13(1):e03822-21. doi: 10.1128/mbio.03822-21 (PMC8844916; doi:10.1128/mbio.03822-21)
Supplement: FIG S2 [file mbio.03822-21-sf002.pdf]

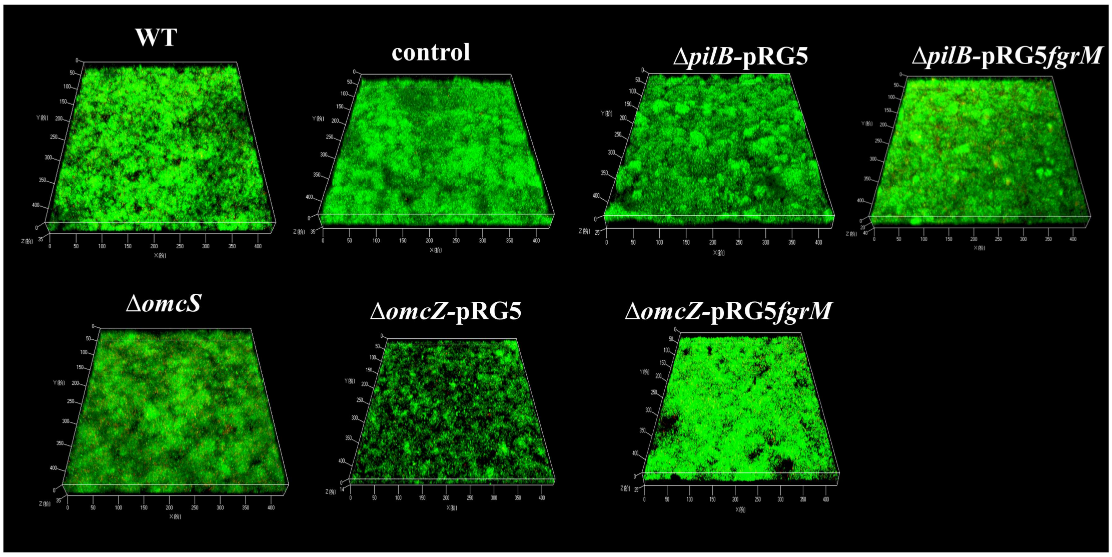

Figure S2. Representative confocal laser scanning microscopy images of anode biofilms from different *G. sulfurreducens* strains.
