# Supplementary material for: Dissecting the Structural and Conductive Functions of Nanowires in Geobacter sulfurreducens Electroactive Biofilms
Source: mBio. 2022 Feb 15;13(1):e03822-21. doi: 10.1128/mbio.03822-21 (PMC8844916; doi:10.1128/mbio.03822-21)
Supplement: FIG S3 [file mbio.03822-21-sf003.pdf]

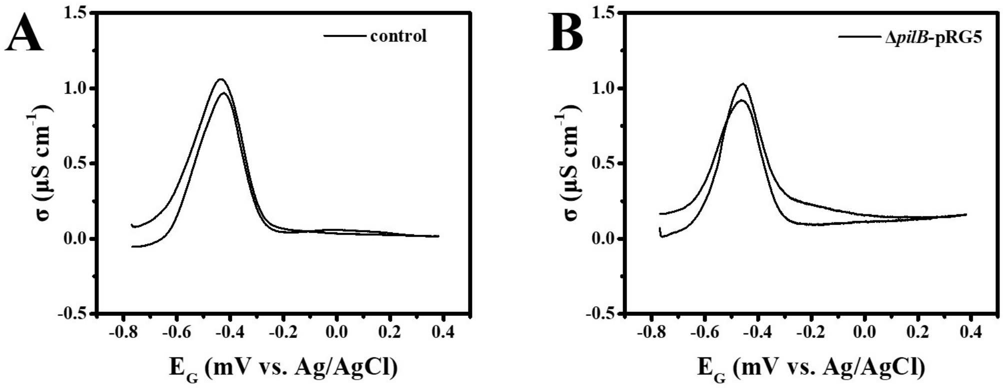

Figure S3. Calculated conductivity versus gate potential response of *G. sulfurreducens* strains (A) control and (B) Δ*pilB*-pRG5 biofilms. The electrochemical gating measurements were directly performed on interdigitated microelectrode arrays (IDAs) using a biopotentiostat (CHI760E, CH Instruments, Inc.). The IDAs had a double-band configuration consisting of 130 antiparallel gold rectangular microelectrodes that had dimensions 2 mm long × 10 μm wide × 90 nm thick and were set apart by 5 μm wide gaps. Potentials with a fixed offset bias of 10 mV (V_{SD}) were applied on IDAs and scanned between −0.6 and 0.3 V with a slow rate of 1 mV s^{-1} simultaneously. The same measurement was also performed with an offset bias of 0 mV to calculate the total background current, which was subtracted from the current measured at a bias of 10 mV, generating the source and drain current at the individual gate potential (E_G). The conductivity (σ) was calculated following the equation: $\sigma = \frac{I_{SD}}{S \times V_{SD}}$, scaling factor $= \frac{1}{S \times V_{SD}} =$ 4.9 cm^{-1} V^{-1}
