# Supplementary material for: Dissecting the Structural and Conductive Functions of Nanowires in Geobacter sulfurreducens Electroactive Biofilms
Source: mBio. 2022 Feb 15;13(1):e03822-21. doi: 10.1128/mbio.03822-21 (PMC8844916; doi:10.1128/mbio.03822-21)
Supplement: FIG S5 [file mbio.03822-21-sf005.pdf]

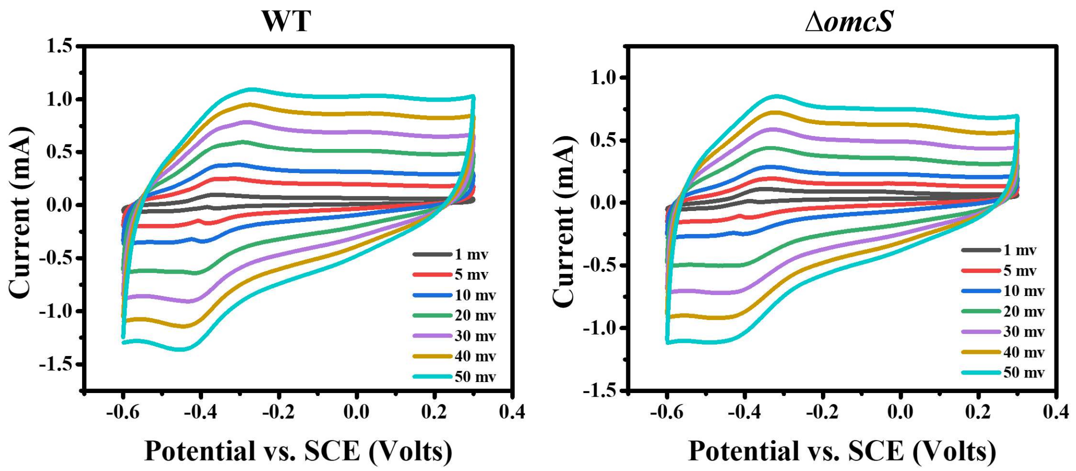

Figure S5. Cyclic voltammograms at increased scan rates under nonturnover conditions for *G. sulfurreducens* WT strain and strain Δ*omcS* biofilms. Cyclic voltammetry was performed in situ under nonturnover conditions by scanning the biofilm electrode from 0.3 to -0.6 V.
