# Supplementary material for: Dissecting the Structural and Conductive Functions of Nanowires in Geobacter sulfurreducens Electroactive Biofilms
Source: mBio. 2022 Feb 15;13(1):e03822-21. doi: 10.1128/mbio.03822-21 (PMC8844916; doi:10.1128/mbio.03822-21)
Supplement: FIG S6 [file mbio.03822-21-sf006.pdf]

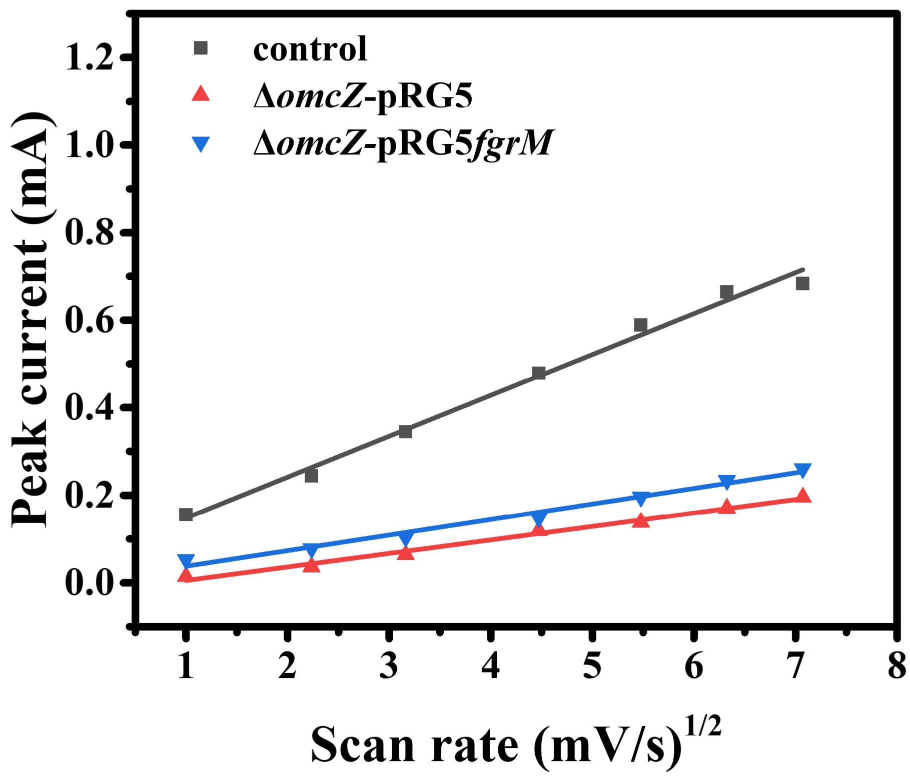

Figure S6. Linear dependence of baseline-subtracted oxidation peak current height in cyclic voltammogram with the square root of the scan rate.
