# Supplementary material for: Dissecting the Structural and Conductive Functions of Nanowires in Geobacter sulfurreducens Electroactive Biofilms
Source: mBio. 2022 Feb 15;13(1):e03822-21. doi: 10.1128/mbio.03822-21 (PMC8844916; doi:10.1128/mbio.03822-21)
Supplement: FIG S7 [file mbio.03822-21-sf007.pdf]

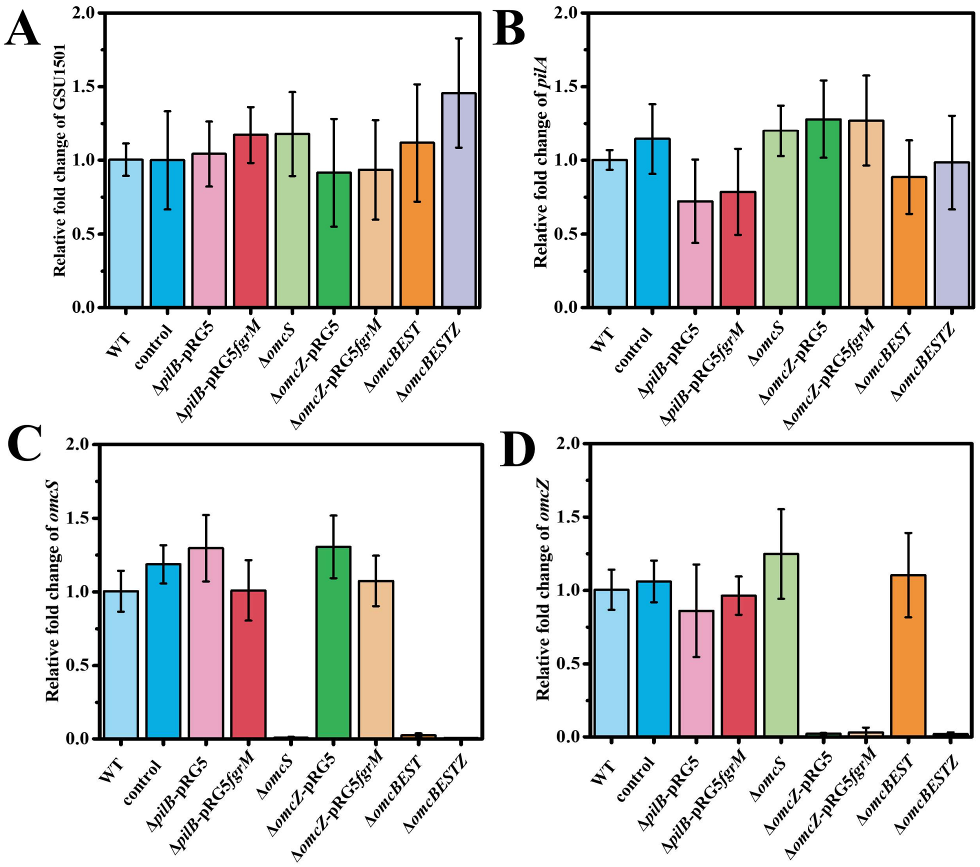

Figure S7. Fold changes in the relative expression profiles of the GSU1501 (A), *pilA* (B), *omcS* (C) and *omcZ* (D) genes in the *G. sulfurreducens* wild-type strain (WT), control strain and mutant strains of Δ*pilB*-pRG5*,* Δ*pilB*- pRG5*fgrM*, Δ*omcS*, Δ*omcZ*-pRG5, Δ*omcZ*- pRG5*fgrM*, Δ*omcBEST* and Δ*omcBESTZ,* respectively. Mean values and standard deviations were obtained from three independent cultures. Statistically significant changes in gene expression were determined using the t-test. The expression of each gene was relative quantified by qPCR normalization against the housekeeping gene *rpoD* (GSU3089).
