# Supplementary material for: Dissecting the Structural and Conductive Functions of Nanowires in Geobacter sulfurreducens Electroactive Biofilms
Source: mBio. 2022 Feb 15;13(1):e03822-21. doi: 10.1128/mbio.03822-21 (PMC8844916; doi:10.1128/mbio.03822-21)
Supplement: TEXT S1 [file mbio.03822-21-s0001.pdf]

Supplementary Text 1

Mutants construction

To construct strain *G. sulfurreducens* Δ*omcZ*, three fragments were prepared firstly: the primer pairs omcZupF/omcZupR and omcZdnF/omcZdnR were used to amplify the sequences 500 bp upstream and 500 bp downstream, respectively, of GSU2076, using *G. sulfurreducens* genomic DNA as a template, and the primer pair gentF/gentR was used to amplify the gentamycin resistance cassette flanked by *loxP* sites from plasmid pCM351. These three fragments were connected with the linear pUC19 plasmid using the In-Fusion HD Cloning Kit, generating plasmid pUC-*omcZ*. This plasmid was linearized with *ScaI* and then electroporated into electrocompetent *G. sulfurreducens* cells. Mutant strain *G. sulfurreducens* Δ*omcZ* was selected on NBAF agar plates containing gentamycin and verified by PCR. Plasmids of pRG5 and pRG5-*fgrM* were transferred into strain Δ*omcZ* further by electroporation, generating strain *G. sulfurreducens* Δ*omcZ*-pRG5 and Δ*omcZ*-pRG5*fgrM* respectively.

To construct strain *G. sulfurreducens* Δ*omcBEST*, three plasmids were prepared firstly: (1) the primer pairs omcBupF/omcBupR and omcBdnF/omcBdnR were used to amplify the sequences 500 bp upstream and 500 bp downstream, respectively, of GSU2737, using *G. sulfurreducens* genomic DNA as a template. These two fragments together with the gentamycin resistance cassette were connected with the linear pUC19 plasmid using the In-Fusion HD Cloning Kit, generating plasmid pUC-*omcB*; (2) the primer pairs omcEupF/omcEupR and omcEdnF/omcEdnR were used to amplify the sequences 500 bp upstream and 500 bp downstream, respectively, of GSU0618, using *G. sulfurreducens* genomic DNA as a template. These two fragments together with the gentamycin resistance cassette were connected with the linear pUC19 plasmid using the In-Fusion HD Cloning Kit, generating plasmid pUC-*omcE*; (3) the primer pairs omcSupF/omcSupR and omcTdnF/omcTdnR were used to amplify the sequences 500 bp upstream and 500 bp downstream, respectively, of GSU2504 and GSU2503 respectively, using *G. sulfurreducens* genomic DNA as a template. These two fragments together with the gentamycin resistance cassette were connected with the linear pUC19 plasmid using the In-Fusion HD Cloning Kit, generating plasmid pUC-*omcST*. These three plasmids were linearized by digestion with *ScaI*. Linearized plasmid pUC-*omcB* was transferred into electrocompetent *G. sulfurreducens* and the resulting cells were selected on NBAF plate containing gentamycin to generate strain Δ*omcB*. To remove the gentamycin cassette from the genome of strain Δ*omcB*, plasmid pCM158 was further transferred into strain Δ*omcB* by electroporation. Colonies were picked from NBAF agar plates containing 200 µg/mL kanamycin and transferred at least three times in liquid NBAF medium without kanamycin. This cell culture was further streaked on a NBAF agar plate without kanamycin and single colonies were picked. These colonies should be sensitive to gentamycin and kanamycin. Removal of gentamycin and kanamycin resistance genes were verified by PCR. Linearized plasmid pUC-*omcE* was further transferred into the gentamycin sensitive OmcB mutant to make double mutation strain. The same procedures were performed to further delete *omcS* and *omcT* genes by transferring linearized plasmid pUC-*omcST*, generating strain Δ*omcBEST*, and

then to delete omcZ gene by transferring linearized plasmid pUC-*omcZ*, generating strain Δ*omcBESTZ.* All mutants were verified by PCR.
