# Supplementary material for: Dissecting the Structural and Conductive Functions of Nanowires in Geobacter sulfurreducens Electroactive Biofilms
Source: mBio. 2022 Feb 15;13(1):e03822-21. doi: 10.1128/mbio.03822-21 (PMC8844916; doi:10.1128/mbio.03822-21)
Supplement: TABLE S1 [file mbio.03822-21-st001.pdf]

Table S1. Bacterial strains and plasmids used in this study

Strain or plasmid	Characteristics	Source or reference
Strains		
E. coli		
DH5α	F^-, φ 80d*lacZ* ΔM15, Δ(*lacZYA -argF*) U169, *deoR* , *recA1* , *endA1* , *hsdR17* (rK^-, mK^+) , *phoA*, *supE44* , λ^-, *thi -1*, *gyrA96* , *relA1*	Takara
G. sulfurreducens		
WT	Strain PCA	1
control	*G. sulfurreducens* PCA carrying plasmid pRG5	Lab stock
GS-Δ*pilB*	Δ*GSU1491::Gm*^{r}	Lab stock
Δ*pilB*-pRG5	Strain GS-Δ*pilB* carrying plasmid pRG5	This work
Δ*pilB*-pRG5*fgrM*	Strain GS-Δ*pilB* carrying plasmid pRG5-*fgrM*	This work
Δ*omcS*	Δ*GSU2504::Gm*^{r}	This work
Δ*omcZ*	Δ*GSU2076::Gm*^{r}	This work
Δ*omcZ*-pRG5	Strain Δ*omcZ* carrying plasmid pRG5	This work
Δ*omcZ*-pRG5*fgrM*	Strain Δ*omcZ* carrying plasmid pRG5-*fgrM*	This work
Δ*omcBEST*	Δ*GSU2737,* Δ*GSU0618,* Δ*GSU2503,* Δ*GSU2504::Gm*^{r}	This work
Δ*omcBESTZ*	Δ*GSU2737,* Δ*GSU0618,* Δ*GSU2503,* Δ*GSU2504,* Δ*GSU2076::Gm*^{r}	This work
Plasmids		
pUC19	In-Fusion Cloning vector; *Amp*^{r}	Takara
pCM158	Cre recombinase expression vector; *Km*^{r}	2
pRG5	Spectinomycin resistance gene, *Sp*^{r}	3

pCM351	Gentamycin resistance cassette flanked by *loxP* sites, *Gm*^{r}	4
pUC-*omcS*	Plasmid pUC19 carrying 500 bp upstream and 500 bp downstream of gene *omcS*; *Amp*^{r}, *Gm*^{r}	This work
pUC-*omcZ*	Plasmid pUC19 carrying 500 bp upstream and 500 bp downstream of gene *omcZ*; *Amp*^{r}, *Gm*^{r}	This work
pUC-*omcST*	Plasmid pUC19 carrying 500 bp upstream of gene *omcS* and 500 bp downstream of gene *omcT*; *Amp*^{r}, *Gm*^{r}	This work
pUC-*omcB*	Plasmid pUC19 carrying 500 bp upstream and 500 bp downstream of gene *omcB*; *Amp*^{r}, *Gm*^{r}	This work
pUC-*omcE*	Plasmid pUC19 carrying 500 bp upstream and 500 bp downstream of gene *omcE*; *Amp*^{r}, *Gm*^{r}	This work
pRG5-*fgrM*	pRG5 carrying *fgrM* gene (KN400_0269), *Sp*^{r}	This work

Ap^r, Ampicillin resistance; *Gm*^{r},Gentamicin resistance; *Sp*^{r} Spectinomycin resistance.

1. Caccavo F, Jr., Lonergan DJ, Lovley DR, Davis M, Stolz JF, McInerney MJ. *Geobacter sulfurreducens* sp. nov., a hydrogen- and acetate-oxidizing dissimilatory metal-reducing microorganism. *Appl Environ Microbiol* **60**, 3752-3759 (1994).
2. Summers ZM, Ueki T, Ismail W, Haveman SA, Lovley DR. Laboratory evolution of *Geobacter sulfurreducens* for enhanced growth on lactate via a single-base-pair substitution in a transcriptional regulator. *ISME J* **6**, 975-83 (2012).
3. Reguera G, McCarthy KD, Mehta T, Nicoll JS, Tuominen MT, Lovley DR. Extracellular electron transfer via microbial nanowires. *Nature* **435**, 1098-101 (2005).
4. Marx CJ, Lidstrom ME. Broad-host-range cre-lox system for antibiotic marker recycling in gram-negative bacteria. *BioTechniques* **33**, 1062-1067 (2002).
