# Supplementary material for: Dissecting the Structural and Conductive Functions of Nanowires in Geobacter sulfurreducens Electroactive Biofilms
Source: mBio. 2022 Feb 15;13(1):e03822-21. doi: 10.1128/mbio.03822-21 (PMC8844916; doi:10.1128/mbio.03822-21)
Supplement: TABLE S2 [file mbio.03822-21-st002.pdf]

Table S2. Primers used for mutant construction and sequence.

Primer name	Amplification/Purpose	Sequence (5' to 3')	Source
omcSupF	GSU2504 upstream	cggtacccggggatcTCGCCGGTCACCGGTAAG	This work
omcSupR		TCCTCCTCGTTATTGTCGGCTTTGTGTCCCCACCTTGCG	This work
omcSdnF	GSU2504 downstream	CCATGGCGGGGAGCGATGACCGCTGCCTACTATGGCCGTA	This work
omcSdnR		cgactctagaggatcGGCACCCAGCTGTAGGTC	This work
gentFor	*Gm*^{r}*-loxp*	CACTAGTAACGGCCGCCAG	This work
gentRev		GGCCGCCAGTGTGATGGAT	This work
omcBupF	GSU2737 upstream	cggtacccggggatcAGCAATTCTGCCTCGACTGTCA	This work
omcBupR		TCCTCCTCGTTATTGGAAACCGCAAGCACAGCAGAAT	
omcBdnF	GSU2737 downstream	CCATGGCGGGGAGCGACTCGACTGGACAGCGCCA	This work
omcBdnR		cgactctagaggatcCTCCTTGTCCGTCGTCCCAT	
omcEupF	GSU0618 upstream	cggtacccggggatcGCCAGACGGATTTGCAGCAG	This work
omcEupR		TCCTCCTCGTTATTGTCAGGTCGTGCTTGGTATTCTTG	
omcEdnF	GSU0618 downstream	CCATGGCGGGGAGCGATGAAGACCGGGGACGTGAC	This work
omcEdnR		cgactctagaggatcTAGCCCAGGGCAAGGTCGTA	

omcTdnF	GSU2503 downstream	CCATGGCGGGGAGCGGCCTCGGCAACGAGTTCATGA	This work
omcTdnR		cgactctagaggatcGGGCATCAGGGAATAGAGGTTGA	
omcZupF	GSU2076 upstream	cggtacccggggatcAGCTTGGCGGAAGAGAACGTCA	This work
omcZupR		TCCTCCTCGTTATTGCGCTGACGTGACACTCGAGAC	
omcZdnF	GSU2076 downstream	CCATGGCGGGGAGCGCTACGAGCTCCGCATCACCAA	This work
omcZdnR		cgactctagaggatcCGAAATCCTACAATGACCTTCCTGT	
fgrMf	Amplifying KN400_0269	CCATGGTTACGAATTGTCATGCCGTCAAGGGGAGA	This work
fgrMr		GGATCCCCGGGAATTCTATGCAAGAGACGTGGAAATTAC	
verOmcS-f	omcS mutation verification	GTCTTTCCGTGGCAGCAGCA	This work
verOmcS-r		GAAGCTGGTTGGCCGGATAG	
verOmcB-f	omcB mutation verification	CAACCAACTCGGACACCGCAT	This work
verOmcB-r		CAGTCAGTGTGGATCGTAGCAT	
verOmcE-f	omcE mutation verification	GCCACATTCAAGGCGACGAAC	This work
verOmcE-r		GGGTTGGTGCCGGTATCGA	
verOmcT-f	omcT mutation verification	AGCTACCACATCAGTACCGCA	This work
verOmcT-r		CATCGGCCACGGTCATGAACT	
verOmcZ-f	omcZ mutation verification	CTACAGCGCAAGCTCCGTCA	This work
verOmcZ-r		ACCGAGAAGGGAGTGAGTGTCA	
qGSU2751f	GSU2751 quantification	CCAGCTACGCCTACTTCTTCTTT	This work
qGSU2751r		AAGCTGTGGTTCAGGAGGTATTT	

The sequences with yellow background represent 15 bp overlapping sequences required for In-Fusion cloning.
